# The Application of a Combined Online and Offline Health Intervention Model for Assessing Parents' KAP About Testicular Torsion: A Self‐Controlled Study

**DOI:** 10.1002/nop2.70255

**Published:** 2025-06-12

**Authors:** Qin Xia, Chengli Wu, Yanjun Gou, Ruixia Wang

**Affiliations:** ^1^ Department of Pain Third Affiliated Hospital of Zunyi Medical University (First People's Hospital of Zunyi) Zunyi China; ^2^ School of Nursing Guizhou Medical University Guiyang China; ^3^ Nursing Department Affiliated Hospital of Guizhou Medical University Guiyang China

**Keywords:** attitudes, behaviours, child parents, health education, health interventions, health promotion, knowledge, nursing, testicular torsion

## Abstract

**Aim:**

To assess the efficacy of a combined online and offline health intervention model tailored to the needs of the study population in the levels of knowledge, beliefs and behaviours of parents about testicular torsion.

**Design:**

A cross‐sectional and a self‐controlled study.

**Methods:**

From March to July 2023, a convenience sampling method was employed to select 500 parents of male children from a kindergarten and a primary school in a city in Guizhou Province, China. A pre‐ and post‐intervention survey was conducted utilising a self‐designed questionnaire to assess the parents' existing knowledge, beliefs and behaviours concerning testicular torsion. This survey utilised a design tailored to the study, incorporating both before and after control measures.

**Results:**

A total of 476 children's parents completed a 3‐month health intervention in this study. Knowledge of testicular torsion among children's parents increased from 21.71% before the intervention to 74.85% after the intervention, and the difference was statistically significant ($\chi^{2}$ = 3767.559, *p* < 0.001); children's parents' attitude scores toward testicular torsion increased from (35.72 ± 3.55) before the intervention to (39.84 ± 2.56) after the intervention, and the difference was statistically significant (*t* = 20.193, *p* < 0.001); and the behavioural scores of children's parents toward testicular torsion increased from (15.72 ± 3.02) points before the intervention to (38.08 ± 1.62) points after the intervention, with a statistically significant difference (*t* = 137.454, *p* < 0.001).

**Conclusion:**

The health intervention, combining both online and offline methods, significantly improved parental knowledge of testicular torsion. It also effectively shifted parental beliefs and behaviours toward more proactive management of the condition. This model demonstrates considerable potential for practical implementation of testicular torsion knowledge among parents, thereby potentially reducing critical response times in clinical scenarios.

**Impact:**

The current study highlights the effectiveness of a comprehensive health intervention model that integrates online and offline components in enhancing the knowledge and beliefs of parents regarding testicular torsion. Additionally, it provides novel insights for enhancing parental knowledge and beliefs about testicular torsion, aiming to mitigate prehospital delays and reduce the likelihood of orchiectomy.

**Reporting Method:**

Study methods and results reported in adherence to the STROBE checklist.

**Patient or Public Contribution:**

No patient or public contribution.

## 1 Introduction

The optimal window for salvaging a testicular torsion (TT) is within 6–8 h, as more delays increase the likelihood of testicular resection due to ischemic necrosis (Mellick et al. 2019). A significant factor contributing to delays in prehospital treatment is parents' lack of awareness and vigilance regarding TT (Friedman et al. 2016; Göger et al. 2020). However, parents aware of TT can better seek medical intervention, resulting in a notable reduction in prehospital delays (Ubee et al. 2014; El Anzaoui 2015). The knowledge‐belief‐behaviour theory, a widely employed framework in academic literature, elucidates how individuals' knowledge and attitudes transform and affect their health‐related behaviours. This theory holds a significant influence on behavioural intervention, as acknowledged by the CDC (Yang et al. 2022). Enhancing the knowledge, attitudes and behaviours of parents regarding TT can effectively prevent orchiectomy, minimise prehospital delays and safeguard the reproductive health of children.

### 1.1 Background

TT or spermatic cord torsion, is characterised by twisting one or both sides of the spermatic cord and obstruction of the blood supply to the testes. It may lead to testicular ischemia, hypoxia and necrosis. The prognosis remains unfavourable, necessitating immediate surgical intervention. The initial documentation of this condition can be attributed to Del asiauvein 1840 (Shunmugam and Goldman 2021). Testicular torsion can manifest at any age; however, it exhibits two distinct peak periods during the neonatal and peripubertal periods. It represents a significant genitourinary emergency in boys. It accounts for a substantial proportion of paediatric scrotal diseases, ranging from 13% to 54% (Clement et al. 2022; Karaguzel et al. 2014). In Korea, the corresponding annual incidence rate of TT among males younger than 19 years is 6.99 per 100,000 cases (Choi et al. 2022). Similarly, the incidence rate for males under 25 in the United States is 4.5 per 100,000 cases annually (Greear et al. 2021). In Taiwan and China, the incidence rate for males under 25 is 3.5 per 100,000 cases per year (Huang et al. 2013). Lastly, the incidence rate of TT in Brazil is 1.4 per 100,000 cases (Korkes et al. 2012).

The testis, functioning as both a male sex organ and an endocrine organ, plays a crucial role in the growth and development of boys. Various factors can contribute to the loss of testicles in children, including testicular tumours, TT, congenital testicular hypoplasia, testicular trauma and cryptorchid torsion. However, TT is the primary cause of testicular loss in boys (Clement et al. 2022). Sudden onset and unfavourable prognosis characterise this condition, and the absence of prompt and efficient treatment can result in severe consequences, such as orchiectomy, infertility, depression and legal actions (Aquila et al. 2021; Menzies‐Wilson et al. 2022; Zhang et al. 2020).

Mellick et al. (2019) determined that the optimal timeframe for testicular salvage falls in a range of 6–8 h. Engaging in testicular exploration, repositioning and fixation within this designated period yields a substantial likelihood of successful testicular salvage. However, further delay increases the probability of testicular resection because of ischaemic necrosis. Roberts et al. (2021) determined that the testicular retention rate is 80% among patients who seek medical attention within 6 h of experiencing symptoms. However, this rate decreases to almost 20% and 10% after 12 and 24 h, respectively. Several studies (Steeman et al. 2022; Sugrue et al. 2022; Yu et al. 2021) have indicated that the prevalence of orchiectomy and the frequent failure of patients seeking prompt medical care within the critical 6–8‐h window could be attributed to a lack of awareness and vigilance regarding TT. These factors contribute to delays in prehospital treatment. Consequently, timely medical intervention upon the manifestation of symptoms emerges as a pivotal determinant for the achievement of testicular preservation, with a particular emphasis on aiming to administer treatment within the critical timeframe known as the ‘golden hour’ (6–8 h).

Studies conducted by Friedman et al. (2016) and Göger et al. (2020) have revealed a notable deficiency in parental knowledge and formal education regarding TT, leading to a reluctance to seek medical assistance when their children encounter scrotal or lower abdominal pain. Instead, parents tend to adopt a watchful approach, delaying treatment. Consequently, promoting awareness of TT among parents of children can mitigate the risk of testicular loss. El Anzaoui (2015) reported that 72% of children presented to the hospital within 6 h after the onset of scrotal pain, and 92% had never heard of TT. Similarly, Ubee et al. (2014) indicated that only 22% of parents seek immediate medical attention for their child's scrotal pain, while 96% of parents believe that there should be increased public awareness regarding TT. Parents who knew about TT promptly sought medical assistance, significantly reducing prehospital delays. The knowledge, attitude and behaviour exhibited by parents, who serve as the primary guardians of children, significantly influence their ability to promptly seek medical attention after TT, given the limited independence of children. Consequently, it is imperative to comprehend the prevailing state of knowledge, attitude and behaviour among parents of children affected by TT in China and to improve health interventions.

Previous studies on TT in China have been predominantly retrospective, with limited emphasis on in‐hospital surgical intervention as the primary mode of intervention. Regrettably, this approach often leads to a significant delay in treatment, particularly among children, resulting in a substantial incidence of testicular resection. Currently, there is no study measuring parents' knowledge, beliefs and behaviour regarding TT. To this end, the current study investigated the efficacy of an online and offline health intervention model in enhancing parents' knowledge, beliefs and behaviour concerning TT. Our study aimed to serve as a valuable resource for enhancing parents' knowledge, beliefs and behaviour regarding TT, ultimately reducing the delay in seeking medical attention for this condition.

## 2 Methods

### 2.1 Design

The study was conducted using a cross‐sectional and a self‐controlled survey design.

### 2.2 Aims

The current study investigated the efficacy of a comprehensive health intervention model that integrates online and offline components in enhancing parents' knowledge and belief levels regarding TT in children.

The findings of the current study can serve as a valuable resource for enhancing parents' understanding and beliefs about TT, thereby mitigating the need for orchiectomy, minimising pre‐hospital delays and safeguarding children's reproductive health.

### 2.3 Sample and Participants

Based on the formula for determining the unrestricted total sample size in the present study (Chen et al. 2023), denoted as *n* = *Z*_α/2_^2^ × *p* (1 − *p*)/δ^2^, where *Z*_α_/2 = 1.96 (α = 0.05, two‐tailed) represents the confidence level, and δ = 5% signifies the permissible absolute error. It is essential to note that the value of *p* cannot be ascertained. However, when *p* = 0.5, *p* (1 − *p*) attains its maximum value. Consequently, substituting the maximum value of *p* (1 − *p*) instead of the actual *p* (1 − *p*), the resulting sample size was calculated as *n* = 384.16. Considering the inclusion of the effective recovery rate, the sample size increased by 10%–20% in this study. We expanded the sample size to include 423–461 participants, representing a 10%–20% increase from the initial sample size of 384.16. A convenience sampling technique was adopted to enrol 500 parents of male children from a kindergarten and a primary school in a city in Guizhou Province, China.

The study subjects met the inclusion criteria if they: (1) were parents of boys aged 3–12 years; (2) did not have any mental diseases or communication disorders and could independently complete the questionnaire; (3) provided informed consent and willingly participated in the study. The exclusion criteria were as follows: (1) invalid questionnaires in the cross‐sectional survey; (2) parents of boys who suffered from TT; (3) parents of boys who were medical professionals and (4) those who left the study and who declined to participate in the study. Exclusion criteria for participant withdrawal were as follows: (1) individuals who could not continue participation in the study due to physical or other constraints and (2) individuals who requested to withdraw from the study before its completion.

### 2.4 Interventions Methods

We formed a fed a health intervention team comprising a pediatric surgeon, a surgical nurse practitioner, a postgraduate supervisor in surgical nursing and three postgraduate students.

A health intervention material library was established through a comprehensive process involving team brainstorming, analysis of the results of pre‐existing surveys, adherence to established guidelines and the literature (Clement et al. 2022; Friedman et al. 2016; Göger et al. 2020), and expert consultations in paediatric surgery and urology at a tertiary care general hospital in Guizhou province. The following primary topics were addressed: the definition of TT, early detection methods, recommended actions following the onset of TT, the feasibility of self‐recovery, potential consequences, available treatment options, daily preventive measures and illustrative scenarios.

Development of intervention tools: (1) a health promotion PowerPoint presentation entitled ‘Testicular Torsion: “Egg Sorrow”’; (2) a portable scientific quiz brochure on ‘Testicular Torsion: “Egg Sorrow”’. (3) Eight self‐produced science videos, each approximately 1 min in duration, featuring illustrations and synchronised voice‐overs, which encompass the primary content of the intervention library; (4) Five expert micro‐lesson videos, each approximately two minutes in duration, addressing the aetiology, symptoms and emergency response measures for TT.

The intervention processes were as follows: (1) The researcher focused on two offline PPT lectures during parent‐teacher conferences at the school, each lasting nearly 30 min. At the same time, the researcher distributed one portable science Q&A brochure, and members of the research team provided on‐site guidance. (2) We pushed 2 small videos of science popularisation and 2 videos of experts' microclasses through WeChat groups every week, and repeated the push after the videos were finished until the end of 3 months. The intervention plan is detailed in Table 1.

**TABLE 1:** Schedule of interventions.

Items	Element	Length of time	Timing	Sites
First offline intervention	PowerPoint lectures, on‐site guided science quiz brochures	30 min	When schools have parent‐teacher conferences	Large school conference room
Science video 1	What is testicular torsion?	33 s	WeChat group push twice per week, repeat after the push until the completion of 3 months	on‐line
Science video 2	How to recognise testicular torsion at an early stage?	47 s		
Science video 3	What should we do after testicular torsion?	41 s		
Science video 4	Can you recover from testicular torsion on your own?	33 s		
Science video 5	Consequences of testicular torsion	34 s		
Science video 6	Treatment of testicular torsion	33 s		
Science video 7	Do's and don'ts in daily life	41 s		
Science video 8	Scenario	1 min and 05 s		
Expert micro‐teaching mini‐video 1	Causes of testicular torsion	1 min, 34 s	WeChat group push twice per week, repeat after the push until the completion of 3 months	on‐line
Expert micro‐teaching mini‐video2	How to determine testicular torsion in children	2 min, 12 s.		
Expert micro‐teaching mini‐video3	Presentation of testicular torsion	2 min, 07 s		
Expert micro‐teaching mini‐video4	What to do about testicular torsion	2 min, 03 s		
Expert micro‐teaching mini‐video5	Does a child recover from testicular torsion on its own?	1 min, 38 s		
Second offline intervention	PowerPoint lectures, on‐site guided science quiz brochures	30 min	When schools have parent‐teacher conferences	Large school conference room

### 2.5 Data Collection and Tools

From March to July 2023, data were collected by the researcher and 2 fellows before the intervention. After 3 months of intervention, the self‐developed Child Parents' Knowledge and Beliefs about Testicular Torsion Questionnaire was used to measure the change in the level of knowledge and beliefs about testicular torsion of the child's parents before and after the intervention. A paper‐based version of the questionnaire was utilised for on‐site survey and any invalid responses were excluded from the analysis. The survey comprised 32 items, encompassing three distinct dimensions: knowledge (14 items), attitude (9 items) and behaviour (9 items). The knowledge dimension was assessed through a single‐choice question format, where a correct response was assigned a score of 1, while an incorrect response received a score of 0. The total score for the knowledge dimension ranged from 0 to 14, with higher scores indicating a better understanding of the fundamental aspects of testicular torsion by parents. The attitudinal and behavioural dimensions were assessed using a 5‐point Likert scale.

The attitudinal dimensions were measured using the response options ‘strongly disagree, disagree, generally agree, agree, strongly agree’, which were assigned numerical values ranging from 1 to 5, resulting in a potential score range of 9–45. Similarly, the behavioural dimensions were evaluated using the response options ‘never, seldom, sometimes, often, always’, with corresponding numerical values ranging from 1 to 5, yielding a potential score range of 9–45.

The behavioural dimension was assessed using the rating scale ‘never, seldom, sometimes, often, always’, and was assigned a numerical value ranging from 1 to 5, resulting in a total score ranging from 9 to 45. A higher score indicated a more positive attitude and behaviour of parents toward testicular torsion. The questionnaire demonstrated a Kaiser‐Meyer‐Olkin (KMO) value of 0.927, Bartlett's test of sphericity ($\chi^{2}$) of 3374.586 with a significance level (*p*) less than 0.001. Additionally, the questionnaire exhibited a content validity index (S‐CVI) of 0.86 and an item‐level content validity index (I‐CVI) ranging from 0.80 to 1.00 for each item. Furthermore, the questionnaire demonstrated a high internal consistency with a Cronbach's alpha coefficient of 0.933.

### 2.6 Ethical Considerations

This study was conducted following the principles outlined in the Declaration of Helsinki and was approved by the Ethics Committee of REDACTED in Guizhou Province (2022 No. 749). This study is registered with the Chinese Clinical Trial Registry (Registration number: ChiCTR2500096789). Informed consent was obtained from all participants who could withdraw from the study at any time. The study was conducted anonymously, and stringent measures were taken to ensure the confidentiality of participants' information.

### 2.7 Data Analysis

Data were input utilising Microsoft Excel. The statistical software SPSS 25.0 was employed to conduct the statistical analysis. The normality of data was assessed using the Shapiro–Wilk test. Data with a normal distribution are presented as mean ± standard deviation (x ± s), while categorical data are presented as frequency (*n*) or percentage (%). The chi‐squared test ($\chi^{2}$) was employed to assess the differences in scores for each entry within the knowledge dimension, and to compare the overall knowledge awareness scores between pre‐intervention and post‐intervention phases. Additionally, a paired‐sample *t*‐test was used to evaluate the differences in scores for each entry within the attitude and behavioural dimensions and to determine the total scores between the pre‐intervention and post‐intervention phases. The significance level was set at *α* = 0.05.

### 2.8 Quality Control

#### 2.8.1 Pre‐Intervention

To reduce the dropout rate of the research subjects, a WeChat group was established as a means of communication. The members of the group were responsible for maintaining the group to explain the research subjects once again the purpose, significance and process of this study. To ensure the compliance of the participants before the intervention, members of the team introduced the content of the project to the participants in detail and measured their willingness to participate in this study.

#### 2.8.2 Intervention in Progress

To ensure the compliance of the study subjects, the offline intervention was conducted at the time of parent‐teacher conferences in the school. The online intervention was conducted at the convenience of the study participants to increase their acceptance and the effectiveness of the intervention. For instance, the intervention was conducted after dinner and on weekends. The WeChat group not only pushed out the contents related to testicular torsion but also patiently answered the subjects' concerns about other diseases to increase the frequency of opening and using the WeChat health promotion group. With the help of the school, parents submitted homework to improve the behavioural compliance of the study participants.

#### 2.8.3 After the Intervention

After returning the questionnaire, two researchers double‐checked the data to detect any problems such as incorrectly filled in and omitted items and make up for the deficiencies in a timely manner.

## 3 Results

### 3.1 Basic Information

Before the intervention, 500 questionnaires were distributed and 496 valid questionnaires were collected. The 3‐month health intervention was implemented for 496 study subjects. Since 20 cases withdrew during the study period, 476 cases finally completed this intervention and the failure rate of this study was 4.03%. The general information of the study subjects is detailed in Table 2.

**TABLE 2:** General information on study participants (*n* = 476).

Items	Categorisation	Numbers (*n*)	Component ratio (%)
An only child	Yes	122	25.63
	No	354	74.37
Age (years)	≤ 6	148	31.09
	> 6	328	68.91
Parents	Father	110	23.11
	Mother	366	76.89
Age of parents (years)	≤ 30	94	19.75
	31–40	329	69.12
	> 40	53	11.13
Permanent mission	Municipalities	276	57.98
	Villagers	200	42.02
Family structures	Single parents	16	3.36
	Both parents	240	50.42
	Large family	220	46.22
Level of education	Junior high school and below	198	41.60
	High school/Secondary school	77	16.18
Profession	Professional training college	76	15.97
	Undergraduate and above	125	26.26
	Workers	35	7.35
	Agriculture	112	23.53
	Company staff	44	9.24
	Scientific, educational and cultural personnel	42	8.82
	Cadres of organisations	45	9.45
	Self‐employed	19	3.99
	Freelancer	45	9.45
	Otherwise	134	28.15
Monthly household income ($)	< 3000	115	24.16
	3001–4999	175	36.76
	5000–9999	146	30.67
	> 10,000	40	8.40
Main sources of medical expenses	Residents' medical insurance	394	82.77
	Commercial insurance	16	3.36
	(be) at one's own expense	66	13.87
Having family members in the medical profession	Yes	43	9.03
	No	433	90.97

### 3.2 Pre‐ and Post‐Intervention Comparison of Parents' Knowledge of Testicular Torsion

After the intervention, knowledge of testicular torsion increased compared to before the intervention, and the difference was statistically significant (*p* < 0.05). The entry ‘your child thinks of acute gastroenteritis, appendicitis, etc. as the first thing that comes to mind when he or she has abdominal or scrotal pain’ decreased from 71.85% before the intervention to 28.36% after the intervention (*p* < 0.001). The overall knowledge of parents of children about testicular torsion increased from 21.71% to 74.85% (*p* < 0.001) (Table 3).

**TABLE 3:** Pre‐ and post‐intervention comparison of children's parents' knowledge of testicular torsion (*n* [%]).

Knowledge dimension	Pre‐intervention (*n* = 476)	Post‐intervention (*n* = 476)	$\chi^{2}$	*p*
Have you ever heard of testicular torsion in children?	4 (0.84)	12 (2.52)	4.068	0.044
Testicular torsion is predominantly observed during the neonatal period and among adolescents aged 12–18	23 (4.83)	426 (89.50)	684.593	< 0.001
Higher incidence of testicular torsion during cold seasons or sudden temperature changes	31 (6.51)	379 (79.62)	518.815	< 0.001
Testicular torsion may present with symptoms such as abdominal pain, scrotal pain, nausea and vomiting	212 (44.54)	447 (93.91)	272.282	< 0.001
Testicular torsion can be treated with manipulation or surgery	57 (11.97)	405 (85.08)	509.281	< 0.001
Testicular torsion occurs due to vagal excitation, physical exercise, traumatic events and other external factors	47 (9.87)	335 (70.38)	362.647	< 0.001
When a child experiences abdominal or scrotal pain, the initial differential diagnoses typically include conditions such as acute gastroenteritis or appendicitis	342 (71.85)	135 (28.36)	180.039	< 0.001
Testicular torsion predominantly occurs during sleep or immediately before awakening	34 (7.14)	431 (90.55)	661.610	< 0.001
Testicular torsion may result in significant ramifications, including orchiectomy, infertility, depression and other adverse outcomes	284 (59.66)	448 (94.12)	158.998	< 0.001
The torsion of a single testicle can affect the function of the other testicle	179 (37.61)	414 (86.97)	246.958	< 0.001
Testicular torsion has been found to have a genetic association	62 (13.03)	311 (65.34)	273.305	< 0.001
The diagnosis of testicular torsion is confirmed by ultrasound and surgical exploration	40 (8.40)	442 (92.86)	679.116	< 0.001
The recommended golden time for testicular salvage following testicular torsion is 6–8 h	25 (5.25)	450 (94.54)	758.932	< 0.001
The surgical removal of testicular necrosis is a necessary procedure	107 (22.48)	353 (74.16)	254.557	< 0.001
The overall rate of awareness	1447 (21.71)	4988 (74.85)	3767.559	< 0.001

### 3.3 Pre‐ and Post‐Intervention Comparison of Parents' Attitude Scores Toward Testicular Torsion

After implementing the intervention, there was a significant increase in the attitude scores of all parents toward testicular torsion compared with the pre‐intervention period (*p* < 0.01).

Specifically, the total score for the attitude dimension rose from (35.72 ± 3.55) to (39.84 ± 2.56) before the intervention, with a statistically significant difference (*p* < 0.01) (Table 4).

**TABLE 4:** Comparison of children's parents' attitude scores toward testicular torsion before and after intervention ($\bar{x}\pm s$, points).

Attitude dimension	Pre‐intervention (*n* = 476)	Post‐intervention (*n* = 476)	*t*	*p*
A comprehensive understanding of the disease is crucial to effectively address testicular torsion	4.14 ± 0.66	4.51 ± 0.53	9.539	< 0.001
Healthcare professionals ought to augment health education regarding testicular torsion	4.24 ± 0.60	4.48 ± 0.51	6.546	< 0.001
Untimely treatment of testicular torsion may result in both physical and psychological harm to the child	4.18 ± 0.63	4.41 ± 0.51	5.953	< 0.001
Getting your child to the hospital as soon as he or she develops scrotal pain or abdominal pain is pivotal to saving a testicular torsion	4.32 ± 0.55	4.54 ± 0.50	6.583	< 0.001
I am open to acquiring knowledge regarding testicular torsion comprehensively provided by my healthcare provider	4.24 ± 0.62	4.50 ± 0.50	7.022	< 0.001
I will prioritise the well‐being of my child's reproductive health and, if deemed necessary, actively engage healthcare professionals for assistance	2.00 ± 0.85	4.30 ± 0.62	47.385	< 0.001
It is important to teach your child to recognise the early signs of testicular torsion	4.18 ± 0.6	4.38 ± 0.49	5.408	< 0.001
I would be concerned if my child had testicular torsion	4.32 ± 0.55	4.40 ± 0.49	2.310	0.021
The most dependable sources for acquiring knowledge regarding testicular torsion, its prevention and treatment are radio and television broadcasts, WeChat videos, informational pamphlets and lectures delivered by healthcare professionals	4.11 ± 0.67	4.33 ± 0.56	5.458	< 0.001
Total Attitude Dimension Score	35.72 ± 3.55	39.84 ± 2.56	20.193	< 0.001

### 3.4 Pre‐ and Post‐Intervention Comparison of Parents' Behavioural Scores for Testicular Torsion

After implementing the intervention, testicular torsion‐related behavioural scores of parents exhibited a significant increase compared with their pre‐intervention levels (*p* < 0.01). Specifically, the overall score for the behavioural dimensions significantly (*p* < 0.01) rose from (15.72 ± 3.02) before the intervention to (38.08 ± 1.62), demonstrating a statistically significant difference (as presented in Table 5).

**TABLE 5:** Comparison of children's parents' behavioural scores for testicular torsion before and after intervention ($\bar{x}\pm s$, points).

Practice dimension	Pre‐intervention (*n* = 496)	Post‐intervention (*n* = 476)	*t*	*p*
If my child experiences scrotal pain or abdominal pain, I will promptly seek medical attention at a publicly funded secondary or tertiary healthcare facility	2.95 ± 1.58	4.70 ± 0.50	22.922	< 0.001
I provide my children with education on sexual health based on the guidance received from healthcare professionals	1.87 ± 0.80	4.51 ± 0.54	60.095	< 0.001
I will teach my children to prevent external impact on the perineum	1.91 ± 0.85	4.42 ± 0.55	52.382	< 0.001
Following various experiences, including engaging in group activities and physical education classes, I shall diligently monitor my child's physical well‐being	1.68 ± 0.88	4.54 ± 0.55	58.530	< 0.001
I actively engaged in the training session on testicular torsion conducted by the healthcare organisation	1.25 ± 0.62	4.67 ± 0.48	95.804	< 0.001
I will accompany my children to the hospital for routine medical examinations	2.93 ± 1.07	4.21 ± 0.68	21.809	< 0.001
I have my children with me to learn about and be alert to testicular torsion	1.03 ± 0.19	4.66 ± 0.50	151.100	< 0.001
I learned about the prevention and treatment of testicular torsion through various educational resources such as WeChat mini‐videos, brochures, public forums and microblogging communities	1.08 ± 0.43	4.57 ± 0.53	111.388	< 0.001
I talked to people about testicular torsion before	1.02 ± 0.13	1.81 ± 0.87	19.824	< 0.001
Total behavioural dimension score	15.72 ± 3.02	38.08 ± 1.62	137.454	< 0.001

## 4 Discussion

WeChat, known for its convenience, popularity and interactivity, is prevalently used in the healthcare sector due to the emergence of ‘Internet + healthcare’ services (Hoerster et al. 2022; Hoek et al. 2021; Zheng and Yan 2023). We employed a hybrid approach, including both online and offline methods, to deliver testicular torsion know‐and‐trust health interventions to parents. Our primary objective was to enhance the humanisation of offline medical and health services while ensuring online services' convenience. Additionally, we aimed to diversify interventions, improving the understanding and acceptance among study participants, thus making it a feasible strategy.

The knowledge‐belief‐behaviour theory (Yang et al. 2022) is frequently employed in academic settings to elucidate the mechanisms underlying the transformation of an individual's knowledge, attitudes and subsequent modification of health behaviours. Notably, the Centers for Disease Control and Prevention (CDC) has granted this theory a prominent position among the most impactful behavioural intervention theories. Multiple studies (Friedman et al. 2016; Göger et al. 2020; Ubee et al. 2014) have demonstrated a deficiency in parental awareness and standardised education for testicular torsion. Enhancing parental knowledge about testicular torsion among individuals with children can help reduce the incidence of testicular loss. According to a study conducted by Alsulaimani et al. (2023), only 25.4% of the 394 parents who participated in the survey had prior knowledge of testicular torsion, suggesting a lack of health education about this condition.

Similarly, Alenzi et al. (2023) discovered that 52.2% of the 320 included parents had never been acquainted with testicular torsion. The authors emphasised the necessity of increasing parental awareness regarding the testicular torsion of their offspring. Our findings indicated that the parents' collective knowledge of testicular torsion before the intervention stood at 21.71%. However, following the intervention, this figure significantly rose to 74.85%. Moreover, it is noteworthy that all parents experienced a substantial improvement in their understanding of the fundamental aspects of testicular torsion. In total, 4 (0.84%) of the respondents had heard of testicular torsion in children before the intervention, and 12 (2.52%) of the respondents had heard of it after the intervention, because most of the respondents interpreted the word ‘before’ as before the intervention, while a small proportion of the respondents interpreted the word ‘before’ as after the intervention. The use of ‘before’ and ‘this’ was ambiguous in the questionnaire, so most of the respondents interpreted ‘after’ as after the intervention, and ‘before’ was interpreted as before the intervention. Previous research (Friedman et al. 2016; Göger et al. 2020) has demonstrated that delays in seeking medical care for children can be attributed to parental neglect, as they may fail to recognise the symptoms of testicular torsion when scrotal pain or lower abdominal pain arises.

Consequently, this lack of awareness leads to the unfortunate consequence of missing the critical window of 6–8 h for life‐saving treatment of testicular torsion. This study showed that the knowledge dimension entry ‘The first thing that comes to mind when your child has abdominal or scrotal pain is acute conditions, such as gastroenteritis or appendicitis’, decreased from 71.85% before the intervention to 28.36% after the intervention (*p* < 0.01). The entry ‘The optimal time for testicular resuscitation after testicular torsion is 6–8 h,’ increased from 5.25% pre‐intervention to 94.54% post‐intervention (*p* < 0.01). These findings suggest that online and offline health interventions can increase awareness of testicular torsion among parents, thereby reducing pre‐hospital delays, preventing orchiectomy and protecting children's reproductive health.

Previous studies conducted by Friedman et al. (2016) and Göger et al. (2020) have indicated that parents prefer a watchful waiting approach instead of promptly seeking medical attention when their child encounters scrotal pain or lower abdominal pain. This study demonstrated a significant increase in the total score of the attitudinal dimension toward testicular torsion among parents, from pre‐intervention (35.72 ± 3.56) to post‐intervention (39.84 ± 2.56) (*p* < 0.01). Furthermore, all individual entries significantly increased post‐intervention (*p* < 0.01).

Specifically, the entry ‘Immediate transport to the hospital when your child has critical scrotal or abdominal pain’ exhibited a significant difference before (4.32 ± 0.55) and after intervention (4.54 ± 0.50) (*p* < 0.01). It suggests that online and offline health interventions can promote changes in the attitudes of parents toward testicular torsion and motivate them to consider it a severe issue.

Ubee et al. (2014) indicated that only 22% of parents promptly sought medical attention for their child's scrotal pain, while the majority of hospital visits (72%) occurred 6 h after the onset of such pain. However, parents who received education on testicular torsion displayed a notable reduction in pre‐hospital delays, indicating their ability to seek timely medical intervention. Several studies (Steeman et al. 2022; Sugrue et al. 2022; Yu et al. 2021) have indicated that children exhibit a higher incidence of testicular resection than adults due to their limited autonomy. Additionally, these studies emphasise the importance of early parental recognition to shorten the duration of testicular ischemia and prevent testicular resection. Therefore, enhancing parental awareness about testicular torsion in childhood is imperative, highlighting the need for pre‐hospital health interventions. These findings indicated that the behavioural dimension score of parents significantly increased from (15.71 ± 3.02) before the intervention to (38.08 ± 1.62) after the intervention. All entries demonstrated significant improvement following the intervention (*p* < 0.01). The frequency of taking a child to a public secondary or tertiary hospital immediately in the presence of scrotal pain or abdominal pain significantly increased from pre‐intervention (2.95 ± 1.58) to post‐intervention (4.70 ± 0.50) (*p* < 0.01). It suggests that online and offline health interventions can promote behavioural change toward testicular torsion and promote healthcare‐seeking behaviours among parents.

### 4.1 Limitations

The present study has several limitations. Firstly, using the before‐and‐after control design due to objective constraints does not allow for sufficient control of the confounding factors. The intervention method was a combination of online and offline measures. The online intervention method may have pitfalls, such as poor compliance of some study participants, lack of understanding of the intervention content and communication with the researcher, which may affect the effectiveness of the intervention. The intervention strategies and techniques should be further explored in the future. Secondly, there are limitations in extrapolating the results. Larger sample sizes are needed for further in‐depth studies in the future.

### 4.2 Implications for the Care of Testicular Torsion in Children

This study offers novel insights for enhancing pre‐hospital delays in testicular torsion, preventing orchiectomy and safeguarding the reproductive health of children. In addition to in‐hospital surgical interventions, it is imperative to direct attention to the underlying factors contributing to pre‐hospital delays in treatment. Furthermore, the government, media, health authorities, schools, medical institutions and healthcare professionals must collaborate to enhance health education on testicular torsion. This collaborative effort can augment parental awareness of testicular torsion and prevent pre‐hospital delays caused by inadequate knowledge about such conditions.

## 5 Conclusions

The combined online and offline health intervention model can improve the parents' knowledge of testicular torsion, promote attitudinal and behavioural changes and facilitate parents' application of the knowledge about testicular torsion. It can help the early diagnosis and treatment of testicular torsion to avoid necrosis and prolonged ischaemia, thus reducing pre‐hospital delays, lowering the rate of orchiectomy and protecting the reproductive health of children.

## Author Contributions

Qin Xia made substantial contributions to conception and design, or acquisition of data or analysis and interpretation of data. Chengli Wu involved in drafting the manuscript or revising it critically for important intellectual content. Yanjun Gou agreed to be accountable for all aspects of the work in ensuring that questions related to the accuracy or integrity of any part of the work are appropriately investigated and resolved. Ruixia Wang given final approval of the version to be published. Each author should have participated sufficiently in the work to take public responsibility for appropriate portions of the content.

## Conflicts of Interest

The authors declare no conflicts of interest.

## Data Availability

The data underlying this article will be shared on reasonable request to the corresponding author.
